# Clinical impact and in vitro characterization of *ADNP* variants in pediatric patients

**DOI:** 10.1186/s13229-024-00584-7

**Published:** 2024-01-22

**Authors:** Chuanhui Ge, Yuxin Tian, Chunchun Hu, Lianni Mei, Dongyun Li, Ping Dong, Ying Zhang, Huiping Li, Daijing Sun, Wenzhu Peng, Xiu Xu, Yan Jiang, Qiong Xu

**Affiliations:** 1 Institutes of Brain Science, State Key Laboratory of Medical Neurobiology and MOE Frontiers Center for Brain Science, Fudan University, Shanghai, 200032 China; 2 Department of Child Health Care, Children’s Hospital of Fudan University, Shanghai, 201102 China

**Keywords:** Helsmoortel–Van der Aa syndrome, HVDAS, ADNP syndrome, *ADNP* variants, Autism spectrum disorder, Global developmental delay

## Abstract

**Background:**

Helsmoortel–Van der Aa syndrome (HVDAS) is a rare genetic disorder caused by variants in the activity-dependent neuroprotector homeobox (*ADNP*) gene; hence, it is also called ADNP syndrome. ADNP is a multitasking protein with the function as a transcription factor, playing a critical role in brain development. Furthermore, *ADNP* variants have been identified as one of the most common single-gene causes of autism spectrum disorder (ASD) and intellectual disability.

**Methods:**

We assembled a cohort of 15 Chinese pediatric patients, identified 13 variants in the coding region of *ADNP* gene, and evaluated their clinical phenotypes. Additionally, we constructed the corresponding *ADNP* variants and performed western blotting and immunofluorescence analysis to examine their protein expression and subcellular localization in human HEK293T and SH-SY5Y cells.

**Results:**

Our study conducted a thorough characterization of the clinical manifestations in 15 children with *ADNP* variants, and revealed a broad spectrum of symptoms including global developmental delay, intellectual disability, ASD, facial abnormalities, and other features. In vitro studies were carried out to check the expression of ADNP with identified variants. Two cases presented missense variants, while the remainder exhibited nonsense or frameshift variants, leading to truncated mutants in in vitro overexpression systems. Both overexpressed wildtype ADNP and all the different mutants were found to be confined to the nuclei in HEK293T cells; however, the distinctive pattern of nuclear bodies formed by the wildtype ADNP was either partially or entirely disrupted by the mutant proteins. Moreover, two variants of p.Y719* on the nuclear localization signal (NLS) of *ADNP* disrupted the nuclear expression pattern, predominantly manifesting in the cytoplasm in SH-SY5Y cells.

**Limitations:**

Our study was limited by a relatively small sample size and the absence of a longitudinal framework to monitor the progression of patient conditions over time. Additionally, we lacked in vivo evidence to further indicate the causal implications of the identified *ADNP* variants.

**Conclusions:**

Our study reported the first cohort of HVDAS patients in the Chinese population and provided systematic clinical presentations and laboratory examinations. Furthermore, we identified multiple genetic variants and validated them in vitro. Our findings offered valuable insights into the diverse genetic variants associated with HVDAS.

**Supplementary Information:**

The online version contains supplementary material available at 10.1186/s13229-024-00584-7.

## Background

*D*e novo variants in the activity-dependent neuroprotector homeobox (*ADNP*) gene can result in Helsmoortel–Van der Aa syndrome (HVDAS; OMIM 615873), also referred to as ADNP syndrome [1, 2]. Patients with this syndrome typically exhibit global developmental delay, intellectual disability, and characteristics of autism spectrum disorder (ASD), such as impaired social communication and interaction, repetitive behaviors, and sensory sensitivities [3]. It is estimated that the *ADNP* gene harbors variants in a minimum of 0.17% of genetic cases for ASD, making it one of the most commonly associated genes with ASD [4, 5]. In addition, global developmental delay in these patients often coexists with multi-system dysfunction, primarily manifested as gastrointestinal issues, congenital heart disease, visual impairment, musculoskeletal diseases, and neurological features [6, 7]. Beyond these functional abnormalities, individuals with HVDAS often display specific facial characteristics such as a prominent forehead, high hairline, long philtrum, thin upper lip, and flat nasal bridge [8]. HVDAS is rare but severe syndrome, and little is known with certainty about the prognosis. Treatment is primarily focused on managing symptoms, as no definitive and effective treatment has been clinically established. However, preclinical evidence and pilot studies showed promising effects of Davunetide/NAP, which is the neuroprotective fragment of ADNP and targets the cytoskeleton [9–11]. Furthermore, low-dose ketamine demonstrated to correlate with significant improvements in an open-label study on children with HVDAS [12]. This could potentially be associated with an increase in ADNP expression, but further investigations are required.

The human *ADNP* gene, located on the long arm of chromosome 20 (20q13.13), comprises five exons that encode the activity-dependent neuroprotective protein (ADNP). ADNP featured several structural domains, including nine zinc finger domains, a DNA-binding homeobox domain, an eukaryotic initiation factor 4E (eIF4E) interaction motif, an Alanine–Arginine–Lysine–Serine (ARKS) motif, a heterochromatin protein 1 (HP1) interaction motif (PXVXL), and a nuclear localization sequence (NLS) [13]. Additionally, it included an eight-amino acid peptide segment, known as NAP (NAPVSIPQE), which was considered the neuroprotective fragment of ADNP [14]. NAP had been shown to penetrate the nucleus and ameliorate mutant ADNP-related defects [9, 15]. In embryonic stem cells (ESCs), ADNP interacted with chromodomain helicase DNA-binding protein 4 (CHD4) protein through its N-terminal zinc finger domain, and with the chromo-shadow domain (CSD) of HP1 via its C-terminal PXVXL domain. Together, they formed the ChAHP complex and suppressed transcription by recruiting H3K9me3 [16]. In line with its protein structure, current research showed that ADNP can form local heterochromatin structure and exert transcriptional repression functions [17, 18]. The NLS sequence was crucial for the nuclear distribution of ADNP in cells. While ADNP predominantly localized in the nuclei of various non-neuronal cells from peripheral tissues, it was observed in both the cytoplasm and nucleus of nerve cells in vitro [19]. Animal studies also suggested that ADNP could impact cellular events in the cytoplasm, such as the regulation of cytoskeleton and autophagy [15, 17, 20]. ADNP was shown to bind and stabilize β-catenin, and to play a critical role in maintaining the Wnt/β-catenin signaling during neuronal development [21]. In addition, other studies identified ADNP interaction patterners, including EB1 and EB3, SHANK3 and SIRT1, all critical for the regulation of the cytoskeleton [11, 20, 22].

In recent years, numerous *ADNP* variants reported in patients with clinical manifestations [8, 23–25]. Interestingly, one study classified these variants into two categories based on the location of the mutation in the protein and their status of epigenetic modification: epi-ADNP1, characterized by DNA hypomethylation, with variants primarily located at the N′- or C′- ends of the protein; and epi-ADNP2, characterized by DNA hypermethylation, with variants primarily located at the middle of ADNP protein, notably associated with NLS [26]. Moreover, it was observed that variants at different locations on the protein could influence the various functions of ADNP. For example, frequent variants at p.Y719* in the NLS showed a decreased nuclear/cytoplasmic ratio in neural cells, indicating abnormal nucleocytoplasmic shuttling and associated functions [18, 27]. Notably, a recent study generated a heterozygous animal model carrying the p.Y718* mutation in mouse ADNP, paralog for the human p.Y719* ADNP mutation, thereby effectively replicating the pathology of HVDAS [28]. In N1E-115 cells, the N′-end p.S404* variant significantly impeded microtubule dynamics and assembly, whereas the variant p.E830Efs*83 near the C′-end did not produce the same effect [11]. Therefore, the intrinsic relationship between the complex genotypes and phenotypes of HVDAS is a matter of great interest. Previous study on a cohort of 78 individuals of HVDAS provided limited evidence for the correlation between genotypes and phenotypes [8]. However, a most recent study made substantial efforts to establish the correlation between genotypes and phenotypes of rare genetic diseases including HVDAS by using PhenoScore, a machine-learning framework [29].

In our present study, we compiled a cohort of 15 children diagnosed with HVDAS at the Department of Child Health Care, Children’s Hospital of Fudan University in China, from 2019 and 2022. Genetic variants in the *ADNP* gene were confirmed either through a gene panel or trio-based whole-exome sequencing, conducted either at our institution or at other hospitals. Subsequently, Sanger sequencing validation was performed for all the individuals involved. All the variants potentially affected the coding sequence of *ADNP*. We constructed the corresponding mutant plasmids to examine their expression and subcellular localization in human HEK293T and SH-SY5Y cells. Our study provided a comprehensive overview of the clinical and genetic characteristics of a patient group diagnosed with HVDAS in the Chinese population, contributing valuable insights into this syndrome’s manifestations and its genetic basis.

## Methods

### Clinical evaluations

Clinical evaluations were carried out on children with HVDAS, including a comprehensive medical history assessment across multiple specialties: cardiology, gastroenterology, neurology, ophthalmology, otolaryngology, endocrinology, and the musculoskeletal system. Behavioral issues were noted, and physical examinations were conducted under pediatric supervision. Photographs of the facial features of 13 children were also collected as part of the study. To assess the children's developmental progress and cognitive abilities, two specific tools were used: Griffiths Development Scales-Chinese (GDS-C) [30] and Wechsler Preschool and Primary Scale of Intelligence–Fourth Edition (WPPSI-IV) [31]. GDS-C is a comprehensive tool for assessing the developmental progress of children from birth to 8 years across various areas, including gross and fine motor skills, personal/social abilities, language, and performance. In this study, GDS-C was used to evaluate 12 children. WPPSI-IV is an intelligence test designed to measure cognitive abilities of children aged 2 years and 6 months through 7 years and 7 months, such as language, visuospatial skills, reasoning, working memory, and processing speed. The mean (average) IQ score is set at 100, and the standard deviation (SD) is set at 15. One child in the study was assessed using WPPSI-IV. In addition to these evaluations, auxiliary examinations were performed, including brain magnetic resonance imaging, pelvis and spine x-rays, electroencephalograms, and cardiograms. This study received approval from the Ethics Committee of the Children's Hospital at Fudan University. Images have been published exclusively upon obtaining written informed consent from parents, acting on behalf of their children.

### Plasmid construction

A full-length human *ADNP* (NM_015339.5) expression plasmid was constructed, featuring an HA tag at the N-terminal and both Flag and Myc tags at the C-terminal. In addition, a second open reading frame (ORF) encoding the EGFP protein was placed downstream of the C-terminal tags, separated by the Internal Ribosome Entry Site (IRES) element. This IRES element facilitates cap-independent re-initiation of translation at the second ORF, ensuring that the expression of EGFP was not directly fused to the ADNP protein. Variants were introduced into the same plasmid using the KOD-Plus-Mutagenesis Kit (TOYOBO, SMK-101). The plasmid map, illustrating the aforementioned features, was displayed in Additional file 1: Fig. S1. All primers utilized for cloning are listed in Additional file 2: Table S1. Each plasmid was validated through sequencing, as shown in Additional file 1: Fig. S1.

### Cell culture

HEK293T cells were cultured in DMEM (Gibco, C11995500BT) supplemented with 10% fetal bovine serum (ExCell Biotech, FSP500). SH-SY5Y cells were cultured in DMEM-F12 (Gibco, 11,320,033) supplemented with 10% fetal bovine serum (Thermo, 10,099,141). For transfection, 1 μg of plasmids was introduced into HEK293T cells using Highgene transfection reagent (Abclonal, RM09014). For the SH-SY5Y cells, 1ug of plasmids was transfected using the Lipofectamine™ 3000 transfection reagent (Thermo, L3000001). The cells were cultured for 48 h before immunofluorescence staining, and for 72 h before Western blotting analysis.

### Western blotting

HEK293T cells were harvested and lysed in lysis buffer (2% SDS, 10% glycerol, 0.0625M Tris HCl, pH6.8) supplemented with 1 × protease inhibitor (Beyotime, P1005). The lysates were quantified using the Omni-Easy™ Instant BCA Protein Assay kit (Epizyme Biotech, ZJ102). Equal amounts of each sample were loaded for blotting. The primary antibodies used were: anti-HA (Abmart, M20003S, 1:1000), anti-Flag (Smart lifesciences, SLAB01, 1:5000), and anti-GAPDH (Beijing Ray Antibody Biotech, RM2002, 1:5000). Please see the information of other primary antibodies in Supplementary Methods (Additional file 3). Briefly, the cell lysate and protein marker (Epizyme, WJ103) were electrophoresed on a 10% SDS-PAGE and transferred to a PVDF membrane. After blocking with 5% nonfat milk for 30 min at room temperature, the membrane was incubated overnight at 4 °C with the primary antibodies. After washing with TBST buffer (Tris-buffered saline with 0.1% Tween 20) three times, the membrane was incubated with either an HRP-conjugated goat anti-rabbit (Proteintech, SA00001-2, 1:10,000) or an HRP-conjugated goat anti-mouse (Proteintech, SA00001-1, 1:10,000) antibody for 1 h at room temperature. The signal was developed using an ECL luminescent solution (Tanon, 180-5001) and imaged using a digital chemiluminescence imager.

### Immunofluorescence assay

Both HEK293T and SH-SY5Y cells were cultured on coverslips and fixed with 4% paraformaldehyde for 30 min at room temperature. After washing with PBST (phosphate-buffered saline, 0.1% Triton X-100) three times, the cells were incubated in 1xPBS with 5% normal goat serum and 0.1% Triton X-100 for 30 min at room temperature. The cells were then incubated overnight at 4 °C with primary antibodies: anti-ADNP-C’ (Proteintech, 17987-1-AP, 1:1000) and anti-HA (Abmart, M20003S, 1:1000). After washing with 1xPBS three times, the cells were incubated with secondary antibodies: goat anti-mouse-Fluor 647 (Jackson, 115–605-003,1:1000) and goat anti-rabbit-Fluor Cy3 (Jackson,128–165-160, 1:1000) at room temperature for 1 h in dark room. The nuclei were counterstained with DAPI (Thermo, TC2546141, 1:10000). Following two washes with 1xPBS, the coverslips were mounted on slides using an anti-fade mounting solution (Beyotime, P0126). The images were captured using a Nikon fluorescent microscope.

### Crystal structure modeling

The PDB file, which included the crystal structure information of the human ADNP protein as predicted by the AlphaFold Monomer v2.0 pipeline, was obtained from the open-source UniProt database (Q9H2P0). The 3D structure of human ADNP was subsequently visualized using ChimeraX v1.5, with its functionally conserved domains and mutations identified in our current study mapped onto it.

## Results

### Clinical characterizations in patients with *ADNP* variants

**Fig. 1:**
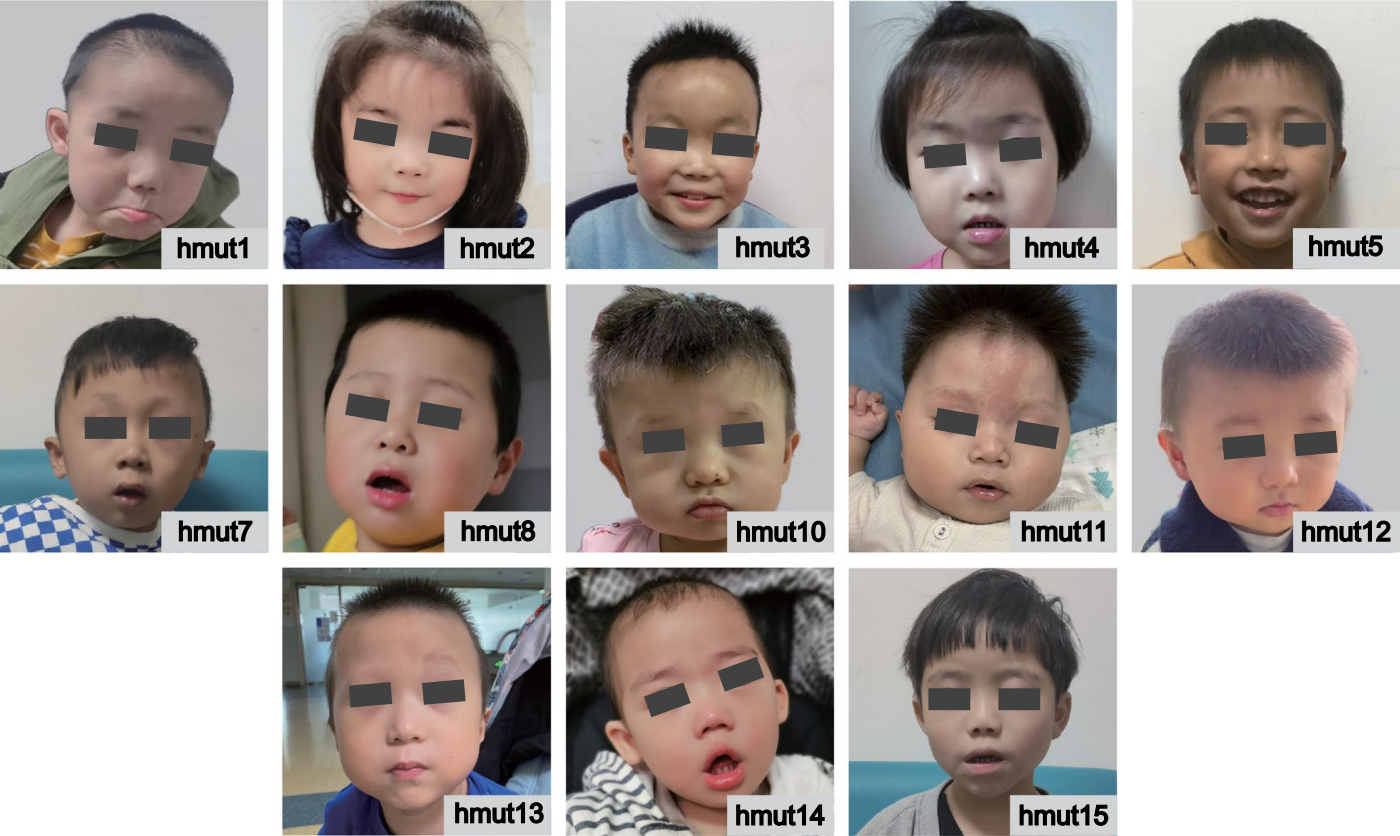
Facial photographs of patients. Noted for a prominent forehead, high hairline, long flat philtrum, thin upper lip, and a flat nasal bridge

To better understand the phenotypic spectrum associated with variants in the *ADNP* gene, we conducted a detailed study of 15 children (hmut1-hmut15), consisting of six girls and nine boys (Table 1 and Additional file 4: Table S2). Facial dysmorphism was common among 13 children (13/13), characterized by features such as a prominent forehead, high hairline, long flat philtrum, thin upper lip, and a flat nasal bridge (Fig. 1). The clinical manifestations are summarized in Table 2. Three out of 15 children were born prematurely. All 15 children exhibited global developmental delay or intellectual disability. Four children displayed features of ASD, characterized by less severe symptoms of social affect, heightened sensory interest, and high levels of stereotyped motor behaviors. Eight children received a clinical diagnosis of ASD according to DSM-5 criteria. Some children had hyperactivity/inattention, aggression/temper tantrums, mood disorders, and self-injurious behavior. Four children had sleep problems, and some showed neurological problems, such as hypertonia, hypotonia, and insensitivity to pain.

**Table 1:** The list of *ADNP* variants identified in the reported individuals

ID	Age/months	Gender	Variant in cDNA (NM_015339.5)	Protein change (Q9H2P0)	Variant type	Inheritance	ACMG	Clinvar	HGMD	SIFT	Polyphen2	Mutationtaster	REVEL	CADD	gnomAD (AC|Hom)
hmut1	42	Male	c.64dupA	p.I22Nfs*3	Frameshift	De novo	Pathogenic (PVS1 + PS2 + PM2 + PP5)	Pathogenic	DM?						0|0
hmut2	38	Female	c.498_499del	p.Y166*	Deletion	De novo	Pathogenic (PVS1 + PS2 + PM2)								0|0
hmut3	86	Male	c.673C > T	p.R225*	Nonsense	De novo	Pathogenic (PVS1 + PM2 + PM6)	Pathogenic	DM			D		35	0|0
hmut5	74	Male	c.2157C > A	p.Y719*	Nonsense	De novo	Pathogenic (PVS1 + PM2 + PM6)	Pathogenic	DM			D		22.9	0|0
hmut6	24	Female	c.2157C > A	p.Y719*	Nonsense	Not available	Pathogenic (PVS1 + PM2 + PM6)	Pathogenic	DM			D		22.9	0|0
hmut7	44	Male	c.2157C > G	p.Y719*	Nonsense	De novo	Pathogenic (PVS1 + PS2 + PM2)	Pathogenic	DM			D		22.9	0|0
hmut8	36	Male	c.2157C > G	p.Y719*	Nonsense	De novo	Pathogenic (PVS1 + PS2 + PM2)	Pathogenic	DM			D		22.9	0|0
hmut10	33	Female	c.2188C > T	p.R730*	Nonsense	De novo	Pathogenic (PVS1 + PS2 + PM2 + PP5)	Pathogenic	DM			D		36	0|0
hmut11	13	Male	c.2188C > T	p.R730*	Nonsense	De novo	Pathogenic (PVS1 + PS2 + PM2 + PP5)	Pathogenic	DM			D		36	0|0
hmut12	16	Male	c.2188C > T	p.R730*	Nonsense	De novo	Pathogenic (PVS1 + PS2 + PM2)	Pathogenic	DM			D		36	0|0
hmut13	42	Male	c.2289delC	p.Y764Mfs*8	Frameshift	Not available	Pathogenic (PVS1 + PS2 + PM2)								0|0
hmut14	25	Male	c.2355_2356 del AA	p.E785Dfs*2	Frameshift	De novo	Likely Pathogenic (PVS1 + PM2)								0|0
hmut15	56	Female	c.2491_2494 del TTAA	p.L831I fs*82	Frameshift	De novo	Pathogenic (PVS1 + PS2 + PM2)	Pathogenic	DM						1|0

**Table 2:** Clinical manifestations of patients with *ADNP* variants in the current study

Clinical features	Percentage (%)	Sample n/Total N	epi-ADNP1*	epi-ADNP2*
ID/GDD	100	15/15	5/5	10/10
Premature birth	20	3/15	2/5	1/10
*Behavioral assessment*				
Autistic features	92.3	12/13	4/5	8/8
Attention-deficit/hyperactivity disorder	100	2/2	1/1	1/1
Temper tantrums/aggression	69.2	9/13	3/5	6/8
Mood disorders	50	1/2	0/1	1/1
Self-injurious behavior	30.8	4/13	1/5	3/8
Sleep problems	30.8	4/13	1/5	3/8
Hypotonia	46.2	6/13	4/5	2/8
Hypertonia	23.1	3/13	0/5	3/8
Insensitivity to pain	46.2	6/13	3/5	3/8
*Multiple systems problems*				
Short stature	13.3	2/15	2/5	0/10
obesity	20	3/15	1/5	2/10
Ophthalmological anomalies	66.7	10/15	5/5	5/10
Cardiovascular anomalies	46.7	7/15	4/5	3/10
Feeding and gastrointestinal problems	66.7	10/15	2/5	8/10
Hearing impairment	13.3	2/15	0/5	2/10
Abnormalities of musculoskeletal system	13.3	2/15	0/5	2/10
Abnormalities of urogenital system	33.3	5/15	0/5	5/10
Seizures	0	0/15	0/5	0/10
Frequent infections	20	3/15	0/5	3/10
Premature eruption of primary teeth	13.3	2/15	2/5	0/10
Widely spaced nipples	20	3/15	2/5	1/10
Hand and foot deformities	53.3	8/15	3/5	5/10
*Neurological findings*				
Mild abnormal brain MRI	100	11/11	4/4	7/7
Abnormal EEG	9.1	1/11	1/4	0/7

Attention-deficit/hyperactivity disorder (ADHD) or mood disorders were only for children 6 years of age and older

*epi-ADNP1 and epi-ADNP2 were categorized according to the episignatures [26]

These children also commonly manifested with multiple system issues, including: (1) Height and Weight: short stature (2/15), obese (3/15) with a body mass index (BMI) above the 97th percentile. (2) Eye Problems: strabismus (6/15), astigmatism (4/15), nystagmus (1/15), and ptosis (1/15). Notably, hmut5 had strabismus and astigmatism, while hmut10 had strabismus and nystagmus. (3) Congenital Heart Conditions: atrial septal defect (5/15) and patent ductus arteriosus (2/15). (4) Feeding and Gastrointestinal Issues: oral movement problems (8/15), frequent vomiting (6/15), and constipation (2/15). (5) Urogenital System Issues: small genitalia (1/15), kidney abnormalities (2/15), cryptorchidism (1/15), and hypospadias (1/15). (6) Musculoskeletal Abnormalities: single palmar crease (2/15), flatfeet (3/15), and mild lateral curvature of fingers (3/15) or toes (3/15), scoliosis (1/15), and hip dysplasia (1/15). (7) Others: hearing impairment (2/15), frequent infections (3/15), early eruption of primary teeth (2/15), widely spaced nipples (3/15), and teeth (2/15).

We further classified all the cases into two groups based on the episignatures described in the literature [26]: epi-ADNP1, which consisted of variants mainly located at the N′- or C′-ends of the protein and is characterized by DNA hypomethylation; and epi-ADNP2, which included variants primarily found in the middle of the ADNP protein (c.2000-2340), particularly associated with NLS, and was characterized by DNA hypermethylation. Notably, hearing impairment, urogenital system abnormalities, and frequent infections were exclusively observed in the epi-ADNP2 group, with proportions of 2/10, 5/10, and 3/10, respectively. In contrast, short stature and premature eruption of primary teeth were solely detected in the epi-ADNP1 group, with each symptom presenting in two out of five cases.

Despite these complications, all of the children maintained normal thyroid function and levels of insulin-like growth factors-1 (IGF-1). Only hmut5 had glucose 6 phosphate dehydrogenase deficiency. Brain magnetic resonance imaging (MRI) revealed mild abnormalities in eleven children (11/11), including wide ventricles, dysplasia of the brain, wide extracerebral space, intracranial cysts, underdevelopment of the corpus callosum, and white matter lesions. One individual exhibited electroencephalogram (EEG) abnormalities (1/11), but none of them experienced seizures (0/15).

### Verification of ADNP expression with identified variations in vitro

**Fig. 2:**
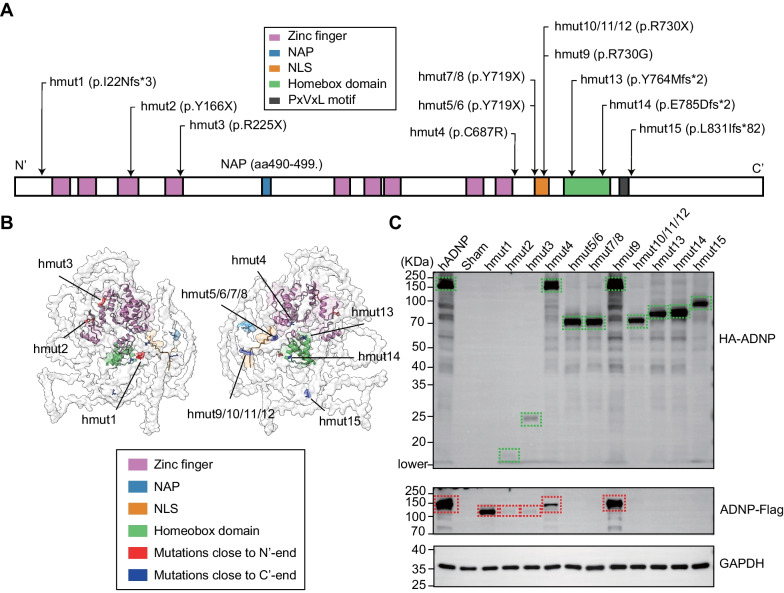
Validation for the expression of *ADNP* mutants in HEK293T cells. **A** Schematic depiction of human ADNP with functional domains indicated, including nine zinc fingers, a NAP (NAPVSIPQ), a nuclear localization signal (NLS), a homeobox domain, and a PXVXL motif. All the mutations were indicated by arrows. Please note that although hmut5/6 and hmut7/8 produce the same effect at the protein level (p.Y719*), they are distinct variants. Specifically, hmut5/6 corresponds to the c2157 C > A mutation, while hmut7/8 corresponds to the c2157 C > G mutation. **B** Predicted 3D structure of human ADNP with all the functional domains and mutations highlighted. **C** Representative Western Blot images showing the expression of wildtype (hADNP) and ADNP mutants (hmuts) in HEK293T cells. Sham, no transfection control. Two different antibodies were used: anti-HA, fused to the N-terminus of ADNP (HA-ADNP); anti-Flag, fused to the C-terminus of ADNP (ADNP-Flag). Dashed lines highlight the main protein bands from anti-HA (green) and anti-Flag (red) blots. GAPDH was used as a loading control. The representative entire blot for anti-Flag was included in the Additional file 6: Fig. S2A

The human *ADNP* gene is composed of five exons, with the start codon located in the third exon [32]. As illustrated in Fig. 2A, it encods a full-length protein of 1,102 amino acids, encompassing multiple functional domains, including nine zinc finger domains at the N-terminus, a NLS, a homeobox domain, and an HP1 interaction motif (PXVXL). Additionally, the NAP sequence (aa490-499) is nestled between the 4th and 5th zinc fingers. In our study, among the 15 cases (hmut1-hmut15) examined, only two (hmut4, hmut9) carried missense variants (Additional file 4: Table S2), which resulted in point mutations in the coding region. The remaining cases exhibited nonsense or frameshift variants, generating new stop codons and producing truncated mutants of varying lengths (Table 1, Additional file 5: Table S3). Notably, hmut5/6, hmut7/8, and hmut10/11/12 shared the mutation at the same location, respectively. We highlighted these variants on the protein map of the full-length human ADNP and observed that their locations either on or in close proximity to the functional domains. As shown in Fig. 2A, hmut1, hmut2, hmut3, and hmut4 were situated on or near the zinc finger domains, suggesting potential interference with the DNA-recognition properties of ADNP. Furthermore, eight cases (hmut5-12) carried four distinct variants at two sites (p.Y719 and p.R730) within the NLS sequence. Clinical cases carrying variants at these two sites reported by multiple groups [18, 33–36], with functional deficits demonstrated in embryonic stem cells [8]. Moreover, hmut13 and hmut14 were situated within the homeobox domain, and it has been reported that deletion of this domain disrupts ADNP’s chromatin binding capacity [33, 37]. Hmut15 was positioned at the C-terminal of the PXVXL motif, known for its interaction with HP1, playing a pivotal role in chromatin packaging and gene silencing [16]. We subsequently visualized the predicted 3D structure of the human ADNP protein (Q9H2P0) using ChimeraX v1.5, and revealed that the functional domains tend to cluster in the center, with the uncharacterized C-terminal region loosely surrounding them (Fig. 2B). Notably, all variants, except for hmut15, were located at the core of the 3D organization of the ADNP protein, where the functional domains congregate (Fig. 2B).

To study the potential deleterious effects of the *ADNP* variants, we constructed expression plasmids containing the full-length human *ADNP* cDNA (*hADNP*), with an HA tag at the N′-end, and both a Myc-tag and a Flag-tag at the C′-end. We also constructed the mutants (hmut1-hmut15), respectively. All plasmids were validated through sequencing (Additional file 1: Fig. S1). Subsequently, we transfected the *ADNP* constructs, including hADNP and 13 mutants, into human HEK293T cells and performed Western blotting (WB) using anti-HA and anti-Flag antibodies (Fig. 2C, Additional file 6: Fig. S2A). As expected, a highly expressed 150KDa band was present in the hADNP group, detectable by both the anti-HA (HA-ADNP) and anti-Flag (ADNP-Flag) antibodies. In addition to the full-length signal, multiple lower-molecular bands were detected in the hADNP group but not in the sham group, suggesting potential protein cleavage or degradation. Similar signals were detected in the groups with the hmut4 and hmut9 missense variants. In addition, we used two different anti-ADNP antibodies: one recognizing the N′-end (aa1-138, ADNP-N′) and the other recognizing the C′-end (aa757-1102, ADNP-C′). Anti-Myc WB was also performed for further validation. The results from the ADNP antibodies were largely consistent with those from antibodies against tags, with the major difference that a faint band around 150KDa was observed in the sham transfection group, which represented the endogenous full-length ADNP protein in HEK293T cells (Additional file 6: Fig. S2).

For all the nonsense and frameshift variants, except for hmut1, truncated mutants of the predicted size (Additional file 5: Table S3) were detected by anti-HA (Fig. 2C) and ADNP-N′ (Additional file 6: Fig. S2D) antibodies, but not by anti-Flag or ADNP-C′ antibody (Fig. 2C, Additional file 6: Fig. S2A–B). Hmut1 carried a frameshift variant predicted to cause early termination at the N′-end. This variant was too short (24aa, 2.6 kDa) to be detected by Western blotting, which could explain why no band was observed for hmut1 when we used both the anti-HA and ADNP-N′ antibodies. The only exception was the endogenous 150 KDa band detected by the ADNP-N′ antibody. It's important to mention that ADNP-N′ (ADNP-F5) is a mouse monoclonal antibody, raised against aa1-138 of human ADNP, and it is expected to recognize both the wildtype ADNP and mutants with an intact N′-end. Surprisingly, when the anti-Flag, anti-Myc or ADNP-C′ antibody was used, a smaller molecular band was observed just below the 150 KDa band (Fig. 2C, Additional file 6: Fig. S2A–C). This band was also detected by the anti-Flag, anti-Myc or ADNP-C' antibody in the hmut2 and hmut3 samples, but not in the other mutants (Fig. 2C, Additional file 1: Fig. S2A–C). However, this unexpected band from hmut1/2/3 was not detected when using anti-HA or anti-ADNP-N′ antibody. This observation suggested a newly formed ADNP protein with an altered N’-terminal, but an intact C′-terminal, detected by in vitro overexpressing hmut1/2/3. Indeed, there is an in-frame ATG site at 229aa of human *ADNP* (NM_015339.5), located downstream of hmut1/2/3 and upstream of all the other mutations.

### Subcellular localization of ADNP mutants in human cells

**Fig. 3:**
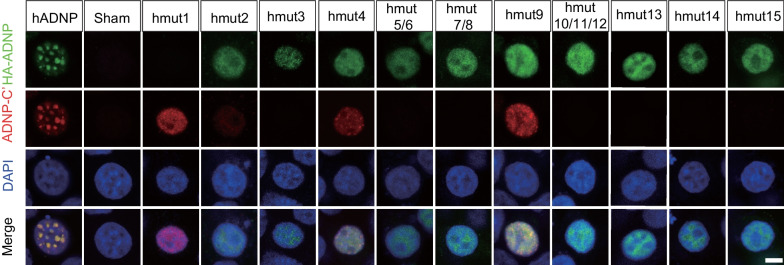
Subcellular location of *ADNP* mutants in HEK293T cells. Representative immunofluorescence images showing the expression pattern of hADNP and ADNP mutants (hmuts) in HEK293T cells. HA-ADNP (green), anti-HA recognizing the HA tag fused to the N-terminus of ADNP. ADNP-C′ (red). Nuclei were counterstained with DAPI (blue). Scale bar, 5 μm

ADNP demonstrated as a transcription factor participating in the formation of local repressive chromatin within euchromatin regions [38]. To explore the subcellular localization of hADNP and all the mutants, we first performed immunofluorescence (IF) staining using anti-HA-tag (HA-ADNP) and ADNP-C′ antibodies in HEK293T cells. IF signals were detected in all samples except for the sham (no transfection) or hmut1 when anti-HA was used (Fig. 3). The endogenous ADNP signal in HEK293T cells was detected, but it was significantly lower compared to the signal from in vitro overexpression (Additional file 7: Fig. S4). In addition, the predicted size of the truncated hmut1 was only 24aa long (Additional file 5: Table S3), which might be too short for stable expression. Interestingly, the ADNP-C′ antibody detected signals not only from hADNP (full-length) and hmut4 and hmut9 (full-length with point mutations), but also from hmut1, further supporting the potential presence of a newly generated isoform in the in vitro overexpressing system (Fig. 3). In the hADNP group, the IF signal from anti-HA and ADNP-C′ antibodies largely overlapped and exhibited a unique subcellular distribution pattern (Fig. 3): (1) the signal was confined to the nuclei and was absent in the cytoplasm, and (2) it formed distinct bodies that were mutually exclusive with the DAPI puncta, suggesting a localized aggregation of hADNP in euchromatin regions. Surprisingly, in all the mutants, the signal remained in the nuclei, including those with variants on the NLS (hmut5-12) (Fig. 3, Additional file 8: Fig. S3). While this finding diverged from previously published data involving 293 T cells, it was important to note that variations in ADNP distribution have been reported across different cell types, states of differentiation in neurons, sex dependency, and in response to the effects of various mutations [18, 33, 39, 40]. Nevertheless, the expression pattern of nuclear bodies was partly or completely disrupted in all ADNP mutants (Fig. 3).

**Fig. 4:**
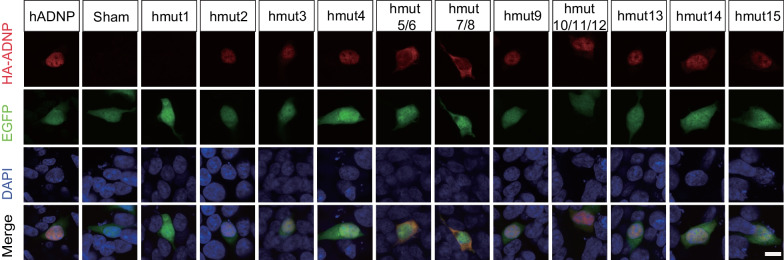
Subcellular location of ADNP mutants in SH-SYSY cells. Representative immunofluorescence images showing the expression pattern of hADNP and ADNP mutants (hmuts) in SH-SY5Y cells. HA-ADNP (red), anti-HA recognizing the HA tag fused to the N-terminus of ADNP. EGFP is expressed as a non-fusion protein that shows the morphology of cells (green). Nuclei were counterstained with DAPI (blue). Scale bar, 10 μm

Drawing from our current research and existing literature, ADNP was primarily localized in the nuclei, though some studies have reported its translocation to the cytoplasm in cultured neuronal cells upon differentiation [27, 41]. To further investigate, we transfected hADNP and mutant constructs in SH-SY5Y cells, a human-derived cell line with neuron-like features. In our ADNP plasmid construct, besides the N’-end and C′-end fused tags, an expressing cassette for EGFP was placed downstream of the C′-end tags, separated by the Internal Ribosome Entry Site (IRES) element. This allowed the EGFP signal to serve as an indicator of successful ADNP transfection, and most immortally, to highlight the morphology of the transfected cells. Similar to what was observed in HEK293T cells, the wildtype ADNP predominantly localized in the nuclei, excluding from DAPI puncta, but forming more nuclear bodies with smaller size (Fig. 4, Additional file 9: Fig. S5). All the mutants remained in the nuclei, with the notable exceptions for hmut5/6 and hmut7/8, which represent two variants of p.Y719* on NLS (Fig. 4). In these two mutant groups, HA-ADNP signal was observed in the cytoplasm, partially in line with a previously published study conducted in a mouse neuroblastoma cell line [18]. Interestingly, another four cases (hmut9, hmut10-12) carrying two variants at p. R730, which is also located on the NLS, showed very little, if any, HA-ADNP signal in the cytoplasm (Fig. 4). This suggests that although the NLS plays a crucial role in the nuclear localization of ADNP in non-differentiated neuronal cells, distinct types of variants may affect the activity of ADNP in different ways.

## Discussion

In current study, we documented 15 cases of patients with *ADNP* variants. Each patient displayed a range of phenotypic features, notably affecting neurodevelopment, cognition, and social functions, which aligns with the clinic features typically observed in HVDAS [1, 8, 26, 36, 42–46]. However, we observed considerable variability in the degree of intellectual capacity, even among individuals with identical *ADNP* variants. For example, among hmut10/11/12, all carrying the variant of p.R730*, hmut12 demonstrated mild, while hmut10 and hmut11 showed severe intellectual disability. Similarly, hmut7/8, carrying the p.Y719* variant, displayed varying degrees of intellectual impairment: moderate for hmut8 displayed, while severe for hmut7. Symptoms in other systems showed significant individual variability as well. Although early tooth eruption has been suggested as a specific feature in previous reports [6], we only observed this characteristic in two of the patients. In our cohort, the incidence of epilepsy and abnormal EEG findings was notably low. None of the patients were diagnosed with epilepsy, and only one child exhibited abnormal EEG results.

In our current study, we categorized all variants into two groups based on their episignatures. Consistent with Dingemans' published article [29], recurrent infections, particularly in the gastrointestinal tract, were observed in the epi-ADNP2 group. However, the children in our epi-ADNP2 group did not exhibit stunted growth, and no apparent neurodevelopmental issues were observed in the epi-ADNP1 group. This discrepancy could be attributed to ethnic differences or potentially be influenced by the small sample size of our study. Future endeavors will focus on gathering more cases to further elucidate the relationship between phenotypes and episignatures in HVDAS. Moreover, an animal model carrying the heterozygous *Adnp* p.Y718* mutation, a paralog of the human *ADNP* mutation p.Y719*, effectively mirrored the pathology of HVDAS [28]. This mouse model exhibited a phenotype similar to that of the *Adnp* haploinsufficient mouse model [47], suggesting at a potential gain of toxic function in the mutant. Interestingly, this study revealed that a lack of positive early life events significantly affected survival rates and amplified abnormal phenotypes. Nonetheless, these adverse effects were partially alleviated with NAP treatment. These findings are highly informative, suggesting that improved parental care in early life could potentially enhance the behavioral outcomes for children diagnosed with HVDAS. Moreover, the *Adnp* haploinsufficient mouse was shown to be more susceptible to stress, with a noted sex difference [48]. Together, these observations imply that while genetic factors are pivotal in HVDAS, non-genetic factors, including both internal and external environmental stressors during developmental, may also participate in the disease's progression. Therefore, in our future clinical endeavors, we plan to provide families with variant at p.Y719* with professional guidance and training to bolster parental care. Additionally, we will carry out long-term follow-up studies to track any potential improvements in these children's behavioral phenotypes.

In our current study, except for hmut4 and hmut9, which are missense variants, all other variants are nonsense mutations that result in loss of function and lead to the production of truncated mutants in an in vitro overexpression system. Both hmut4 and hmut9 carried de novo variants, which was in line with the first missense variant in NAP reported by Dr. Gozes and her colleague [24]. We classified hmut4 and hmut9 as likely pathogenic, adhering to the evidence-based guidelines proposed by the American College of Medical Genetics and Genomics (ACMG) [49]. In future work, we aim to carry out further functional studies to explore the effects of these missense variants. Moreover, all variants were located either within or in close proximity to the functional domains of the *ADNP* gene. Upon examining the predicted 3D model of the ADNP protein, we observed that all the functional domains come together to form the core of the ADNP protein, which is encased by the flexible C′-ends lacking defined structures. Furthermore, it is recognized that multiple regions of ADNP collaborate to form various protein complexes, enabling its multifaceted functions, as seen in the example of ChAHP complex [16, 50]. Therefore, variants at different locations may influence the formation of protein complexes to varying degrees, impacting the protein's function and contributing to the disease phenotype. This could, to some extent, explain the extensive range of genotypes observed in HVDAS, despite the relative consistency in clinical behavioral phenotypes.

There is considerable evidence that hADNP binds to euchromatin and functions as a localized transcriptional repressor [16, 50] [38]. Aline with this, we observed the formation of distinct nuclei bodies in both HEK293T and SH-SY5Y cells upon overexpression of wildtype *hADNP*. However, there were clear differences between these two cell types. Notably, SH-SY5Y cells exhibited a higher number of nuclear bodies, yet these were smaller in size when compared to those in HEK293T cells (Additional file 9: Fig. S5). These differences could be attributed to the distinct cellular contexts of the two cell lines. HEK293T is a widely utilized tumor cell line originating from human embryonic kidney, while SH-SY5Y is a human neuroblastoma cell line. Indeed, original studies on ADNP promoter associations and gene regulation suggested differential ADNP–chromatin interaction in different cell types and upon cellular differentiation [16, 21, 38, 51–53].

In the literature, the mutation of p.Y719* has been extensively studied [8, 18, 24, 26, 39, 54], which was detected in 2 out of 15 cases (p.Y719*, c.2157C>G; p.Y719*, c.2157C>A) in current study. This variant, located on NLS, is a nonsense variant that results in a truncated protein lacking the homeobox domain and HP1-binding motif. Previous studies primarily focused on the impact of this variant on the nucleocytoplasmic distribution of ADNP, considering it the main cause of abnormal cell function like cell proliferation and differentiation [18, 27]. We also observed that ADNP with p.Y719* variant translocated to the cytoplasm in SH-SY5Y cells. Interestingly, another four cases in our study also featured variants (p.R730) located on NLS. Three of these (hmut10/11/12) are nonsense variants (p.R730*) and resulted in truncated mutants as well. hmut9 (p.R730G) is a missense variant, generating a point variant on the ADNP protein. However, none of these variants at p.R730 significantly altered the cytoplasm-nucleus distribution in HEK293T cells. This observation indicates that the disruption of the NLS might only contribute to a portion of the phenotype manifestation. The loss of the functional domain at the C-terminus, or potential anomalies in the 3D structure of ADNP protein complex, could play a pivotal role in mediating the phenotype. Conversely, these mutants that preserve the N-terminal zinc finger structure could exhibit a dominant negative effect, potentially interfering with the functionality of ADNP derived from the normal allele within the patient.

It has been reported that variants near the N-terminus of ADNP resulted in unstable proteins due to the small size of the truncated products [8]. However, in our current study, with the exception of hmut1 (p.I22Nfs*3), the truncated proteins produced by hmut2 (p.Y166*) and hmut3 (p.R225*) were identified using an antibody against the anti-HA at the N-terminal, and an antibody against the N-terminal of ADNP in WB analysis. Additionally, IF staining revealed that these two small mutants were located in the nuclei in both HEK293T and SH-SY5Y cells. This implies that the nuclear localization of ADNP is not strictly regulated by NLS. Some of the zinc finger motifs, retained in these two N'-end truncated mutants, may still have the ability of DNA recognition and binding, thereby maintaining their presence within the nuclei. Despite their nuclear location, the expression pattern of these two mutants significantly differs from that of the wildtype ADNP, suggesting that the absence of other parts of ADNP led to a failure in recruiting other proteins, potentially disrupting the functional chromatin remodeling complex. In addition to these N′-end variants, there are three variants near the C′-end of ADNP were identified in our current study, all of which are frameshift variants: hmut13 (p.Y764Mfs*2*),* hmut14 (p.E785Dfs*2), and hmut15 (p.L831Ifs*82*)*. These variants generate truncated mutants of considerably larger size. While hmut13 (p.Y764Mfs*2) and hmut14 (p.E785Dfs*2) are located within the homeobox domain of ADNP, hmut15 (p.L831Ifs*82) is located downstream of the PXVXL motif, leaving all currently known functional domains unaffected. IF results showed that these mutants remain in the nuclei but lose the punctate distribution in HEK293T cells, suggesting functional disruption. Furthermore, patients carrying these variants still present typical clinical manifestations of HVDAS, implying that the unstructured C′-end of ADNP may also be important in maintaining the active form of ADNP.

In this study, we investigated *ADNP* variants in 15 patients, identifying 11 variants that affected protein expression and intracellular localization. These variants, located within or near functional domains of ADNP, potentially destabilizing the 3D structure, which explains the generally consistent main clinical symptoms across patients. Our study provides valuable insights into the molecular and cellular effects of various *ADNP* variants, highlighting the importance of not only the functional domains but also the unstructured the regions of protein, which might be a crucial factor to consider in future understanding of HVDAS and its treatment design.

## Limitations

Our study provided a cross-sectional view of the patients, so there was a lack of longitudinal studies to track the progression of symptoms and conditions over time. In addition, our in vitro validation in human cell lines might not completely mimic the behavior of neurons in the human brain. Future research should focus on understanding the functional effects of *ADNP* variations in vivo.

## Conclusions

We offered new insights into the clinical features of children with *ADNP* variants and confirmed the detrimental effects of these variants in vitro. Despite the limitations, our findings shed light on the clinical and molecular aspects of HVDAS.

### Supplementary Information

**Additional file 1**. **Figure S1**: Construction and sequencing validation of the human ADNP plasmid for each mutant. (A) Map of the human ADNP plasmid. The open reading frame (ORF) of hADNP is derived from NM015339.5. (B) Featured elements on the hADNP plasmid. Note that an HA tag is situated at the N-terminus, while a Flag and a Myc tag are placed at the C-terminus of hADNP. Additionally, an EGFP ORF is positioned downstream of the tagged ADNP and is separated by an IRES element. (C) Sequencing validation results for the mutation sites of each constructed mutant (hmuts).

**Additional file 2**. **Table S1**: Primer list.

**Additional file 3**. **Supplementary Methods**: Additional primary antibodies used in Figure S2.

**Additional file 4**. **Table S2**: The list of missense variants identified in the reported individuals.

**Additional file 5**. **Table S3**: Predicted protein size corresponding to ADNP mutants.

**Additional file 6**. **Figure S2**: Validation for the mutant proteins with different antibodies in HEK293T cells. Representative Western Blot images showing the expression of wildtype (hADNP) and ADNP mutants (hmuts) in HEK293T cells using antibodies against (A) HA-tag and Flag-tag, (B) ADNP-C’ (aa757-1102), (C) Myc-tag, and (D) ADNP-N’ (aa1-138). GAPDH was used as a loading control. Sham, no transfection control.

**Additional file 7**. **Figure S4**: Endogenous ADNP expression in HEK293T cells. Representative immunofluorescence images showing the expression of ADNP and HA-ADNP in Sham and hADNP groups in HEK293T cells. ADNP-C’ (red), recognizes the C-terminus of ADNP. HA-ADNP (green), recognizes an HA tag fused to the N-terminus of ADNP. Nuclei were counterstained with DAPI (blue). Scale bar, 10 μm.

**Additional file 8**. **Figure S3**: Subcellular localization of ADNP mutant p.Y719X in HEK293T cells. Representative immunofluorescence images showing the expression of hADNP and hmut5/6 (c2157 C>A) and hmut7/8 (c2157 C>G) in HEK293T cells at low magnification. HA-ADNP (purple), anti-HA recognizes an HA-tag fused to the N-terminus of ADNP. ADNP-C’ (red), anti-ADNP recognizes the C-terminus of ADNP. EGFP is expressed as a non-fusion protein on a plasmid that displays cell morphology (green). Nuclei were counterstained with DAPI (blue). Scale bar, 30 μm.

**Additional file 9**. **Figure S5**: Characterization of hADNP-mediated nuclear bodies in HEK293T and SH-SY5Y cells. (A) Representative immunofluorescence images showing the hADNP signal when overexpressed in HEK293T and SH-SY5Y cells. HA-ADNP, HA-tag fused to the N-terminus of ADNP. Nuclei were counterstained with DAPI (blue). Scale bar, 5 μm. (B-C) Statistical analysis of the number (B) and size (C) of nuclear bodies in HEK293T and SH-SY5Y cells. N=12. Mean±SEM. Student’s t test. *P<0.05. #P<0.001.

## Data Availability

All data generated or analyzed during this study are included in this published article.

## Abbreviations

ASD: Autism spectrum disorder
*ADNP*: Activity-dependent neuroprotector homeobox
ADNP: Activity-dependent neuroprotective protein
BMI: Body mass index
eIF4E: Eukaryotic initiation factor 4E
ESCs: Embryonic stem cells
GDS-C: Griffiths development scales-Chinese
HVDAS: Helsmoortel–Van der Aa syndrome
HP1: Heterochromatin protein 1
IGF-1: Insulin-like growth factors-1
IF: Immunofluorescence staining
MRI: Magnetic resonance imaging
NLS: Nuclear localization sequence
WPPSI-IV: Wechsler preschool and primary scale of intelligence-fourth edition
WB: Western blotting
