# Supplementary material for: Clinical impact and in vitro characterization of ADNP variants in pediatric patients
Source: Mol Autism. 2024 Jan 22;15:5. doi: 10.1186/s13229-024-00584-7 (PMC10804707; doi:10.1186/s13229-024-00584-7)
Supplement: Supplementary file 2 — Additional file 2. Table S1: Primer list. [file 13229_2024_584_MOESM2_ESM.docx]

|  | **Primer name** | **Sequence** |
| --- | --- | --- |
| **Primers for HA-hADNP subcloning** | | |
| pCAG-hADNP | EcoR1-1F | ACTGAATTCGCCACCATGTACCCATACGATGTTCCAGATTACGCTTTCCAACTTCCTGTCAACAA |
|  | Xma1-2R | TGCCACCCGGGATCTGTTCAGGAA |
| **Primers for point mutagenesis** | | |
| hmut1 | hmut1-1F | AAAAAAAATACTTAGTGACATTGGGTTGG |
|  | hmut1-1R | CACAGTTTTCCGGGCTTTTCTT |
| hmut2 | hmut2-2F | CTGTTTATTAGTAAGAAGTGCACTTACC |
|  | hmut2-2R | CTTGCTCTACACTGTCA |
| hmut3 | hmut3-3F | GTATTCACTGCAAGTGATGCCTTTTCA |
|  | hmut3-3R | TACTCTCTTCTCGGGCATTCGA |
| hmut4 | hmut4-4F | CATCTAGTTCACCGCAGGGGCGTTGG |
|  | hmut4-4R | CAGAGTGATAGTTGAGGCGGTC |
| hmut5/6 | hmut5-5F | CGCACTTAAGAGCAAATGGAATTTCCC |
|  | hmut5-5R | CTTCACAGGTGCCAGACTT |
| hmut7/8 | hmut7-7F | CGCACTTAGGAGCAAATGGAATTTCCC |
| hhmut9 | hmut9-9F | CCTTACTGAAAAAAGGAAAGTTAGATGATG |
| hmut10/11/12 | hmut10-10F | CCTTACTGAAAAAATGAAAGTTAGATGATG |
|  | hmut10-10R | GAAATTCCATTTGCTCGTAAGT |
| hmut13 | hmut13-13F | GGTCATGAAGATGATTCTATGAAGCCAG |
|  | hmut13-13R | CTTGGGGTCTAAAGCTAAAAC |
| hmut14 | hmut14-14F | CAGGAGAGATTGAGAAGCTA |
|  | hmut14-14R | GTGGGATAGGGCTGTTTGTT |
| hmut15 | hmut15-15F | CATGAAAGAAATAAAGTCAAGC |
|  | hmut15-15R | TTAAACCCCAGCAACACGCC |
| **Primers for sequencing validation** | | |
| hmuts | N’-seq1F | CAACGTGCTGGTTATTGTGC |
|  | hADNP-seq2F | TTGCCCCTACTGTACCTTCA |
|  | hADNP-seq3F | GCTCCCAAACCTCAAGACAA |
|  | hADNP-seq4F | ACATAAAGCTGAGAAAGTCCCAG |
|  | hADNP-seq5F | GTGGTAGCCCTTTTGACCCT |
|  | hADNP-seq6F | ACCAAGTTATTCAGACGGTTCA |
|  | C’-seq1R | CCTCACATTGCCAAAAGACG |

**Table S1: Primer list.**
