# Supplementary material for: Clinical impact and in vitro characterization of ADNP variants in pediatric patients
Source: Mol Autism. 2024 Jan 22;15:5. doi: 10.1186/s13229-024-00584-7 (PMC10804707; doi:10.1186/s13229-024-00584-7)
Supplement: Supplementary file 3 — Additional file 3. Supplementary Methods: Additional primary antibodies used in Figure S2. [file 13229_2024_584_MOESM3_ESM.docx]

**Supplemental Material**

**Supplemental Methods**

**Western blotting**

The entire procedure was detailed in the main text. Additional primary antibodies were used, including: anti-ADNP-N’ (ADNP F-5, Santa Cruz, sc-393377X, 1:1000), anti-Myc ( Proteintech, 60003-2-lg, 1:1000), anti-ADNP-C’ (Proteintech, 17987-1-AP, 1:2000), and anti-GAPDH (Beijing Ray Antibody Biotech, RM2002, 1:5000).
