# Supplementary material for: Clinical impact and in vitro characterization of ADNP variants in pediatric patients
Source: Mol Autism. 2024 Jan 22;15:5. doi: 10.1186/s13229-024-00584-7 (PMC10804707; doi:10.1186/s13229-024-00584-7)
Supplement: Supplementary file 4 — Additional file 4. Table S2: The list of missense variants identified in the reported individuals. [file 13229_2024_584_MOESM4_ESM.docx]

| **ID** | **Age/**  **months** | **Gender** | **Variant in cDNA** | **Protein change** | **Variant type** | **Inheritance** | **ACMG** | **Clinvar** | **HGMD** | **SIFT** | **Polyphen2** | **Mutationtaster** | **REVEL** | **CADD** | **gnomAD** |
| --- | --- | --- | --- | --- | --- | --- | --- | --- | --- | --- | --- | --- | --- | --- | --- |
|  |  |  | **(NM_015339.5)** | **(Q9H2P0)** |  |  |  |  |  |  |  |  |  |  | **(AC\|Hom)** |
| hmut4 | 65 | female | c.2059T>C | p.C687R | missense | *De novo* | Likely pathogenic  (PS2+PM2) | VUS | . | D | D | D | 0.671 | 28.4 | 0\|0 |
| hmut9 | 24 | female | c.2188C>G | p. R730G | missense | *De novo* | Likely Pathogenic  (PS2+PM2) | . | . | D | B | D | 0.195 | 22.3 | 0\|0 |

**Table S2: The list of missense variants identified in the reported individuals.**
