# Supplementary material for: Clinical impact and in vitro characterization of ADNP variants in pediatric patients
Source: Mol Autism. 2024 Jan 22;15:5. doi: 10.1186/s13229-024-00584-7 (PMC10804707; doi:10.1186/s13229-024-00584-7)
Supplement: Supplementary file 5 — Additional file 5. Table S3: Predicted protein size corresponding to ADNP mutants. [file 13229_2024_584_MOESM5_ESM.docx]

| **ID** | **Mutation site** | | **Predicted protein size (aa)** | **Predicted molecular weight (KDa)** |
| --- | --- | --- | --- | --- |
|  | **cDNA (NM015339.5)** | **Protein (Q9H2P0)** |  |  |
| hmut1 | 64 | 22 | 23 | 2.6 |
| hmut2 | 498-499 | 166 | 165 | 19.1 |
| hmut3 | 673 | 225 | 224 | 25.8 |
| hmut4 | 2059 | 687 | 1102 | 123.6 |
| hmut5/6 | 2157 | 719 | 718 | 79.8 |
| hmut7/8 | 2157 | 719 | 718 | 79.8 |
| hmut9 | 2188 | 730 | 1102 | 123.6 |
| hmut10/11/12 | 2188 | 730 | 729 | 81.2 |
| hmut13 | 2289 | 764 | 770 | 85.8 |
| hmut14 | 2355-2356 | 785 | 785 | 87.8 |
| hmut15 | 2490-2494 | 831-832 | 911 | 102.6 |

**Table S3**: **Predicted protein size corresponding to ADNP mutants.**
